# Case-based Surveillance of Influenza Hospitalizations during 2004–2008, Colorado, USA

**DOI:** 10.3201/eid1506.081645

**Published:** 2009-06

**Authors:** Rosemary Proff, Ken Gershman, Dennis Lezotte, Ann-Christine Nyquist

**Affiliations:** University of Colorado Denver, Colorado, USA (R. Proff, D. Lezotte, A-C. Nyquist); Colorado Department of Public Health and Environment, Denver (K. Gershman)

**Keywords:** Influenza, surveillance, hospitalization, Colorado, viruses, research

## Abstract

Colorado became the first state to make laboratory-confirmed influenza-associated hospitalizations a case-based reportable condition in 2004. We summarized surveillance for influenza hospitalizations in Colorado during the first 4 recorded influenza seasons (2004–2008). We highlight the similarities and differences among influenza seasons; no 2 seasons were entirely the same. The 2005–06 influenza season had 2 distinct waves of activity (types A and B), the 2006–07 season was substantially later and milder, and 2007–08 had substantially greater influenza B activity. The case-based surveillance for influenza hospitalizations provides information regarding the time course of seasonal influenza activity, reported case numbers and population-based rates by age group and influenza virus type, and a measure of relative severity. Influenza hospitalization surveillance provides more information about seasonal influenza activity than any other surveillance measure (e.g., surveillance for influenza-like illness) currently in widespread use among states. More states should consider implementing case-based surveillance for influenza hospitalizations.

## Abstract 2

Case-based surveillance provides more information than any other influenza surveillance component.

Each year, in the United States, influenza infections cause an estimated 36,000 deaths (*1*) and >200,000 hospitalizations (*2*). The Centers for Disease Control and Prevention (CDC) monitors seasonal influenza activity to document the timing and geographic spread of influenza infection, track influenza related illness in the community, monitor the proportion of deaths caused by pneumonia and influenza, determine which influenza viruses are circulating, and identify emerging virus changes (*3*). Influenza surveillance can also indicate the relative severity of a given influenza season compared with previous seasons.

Similarly, state health departments monitor seasonal influenza activity within their jurisdictions and contribute data to CDC. There is little standardization, however, among these surveillance systems. All 50 states monitor influenza-like illness (ILI) and report these data weekly to CDC (L. Brammer, pers. comm.), but other measures of influenza activity are conducted only by subsets of states.

In 2004, Colorado became the first state to make influenza-associated hospitalizations a reportable condition and part of routine influenza surveillance (*4*). We summarized the first 4 seasons (2004–2008) of case-based surveillance for influenza hospitalizations in Colorado.

## Methods

Notifiable conditions in Colorado have specified time frames for reporting, either within 24 hours or 7 days of diagnosis. Influenza-associated hospitalizations must be reported within 7 days. The list of notifiable conditions, which includes specified time frames for reporting, is sent to acute care hospitals annually. These conditions are reported primarily by hospital infection control practitioners and may be reported through the Colorado State Health Department’s web-based electronic disease reporting system (CEDRS) or by fax or phone. Colorado has 59 nonfederal acute care hospitals licensed for >25 beds; hospitals licensed for <25 beds (as well as some licensed at 25 beds) are critical access hospitals in rural areas, which infrequently diagnose notifiable conditions.

Data from hospitalized patients with influenza reported to CEDRS for the 2004–2008 influenza seasons are analyzed in this report. Colorado defines an influenza-associated hospitalization as a hospital admission accompanied by a report of an appropriate positive laboratory test result for influenza (*4*). Acceptable and available laboratory tests in Colorado are viral culture, reverse transcription–PCR (RT-PCR), direct immunofluorescent antibody (DFA) staining, and rapid diagnostic tests.

Our analysis defined each influenza season as October 1 through May 31 of the subsequent year. Week 1 (first calendar week) was defined as the week containing January 1 and ending with Saturday of that week. The last week (week 52) corresponded to the last full calendar week of the year that did not contain January 1 of the subsequent year. Specimen collection date was used as a surrogate for date of diagnosis (typically the same date for rapid influenza testing and DFA) or, if not available, the report date. Timeliness of reporting was calculated as the difference between specimen collection date and entry date in CEDRS.

For all 4 influenza seasons, hospitalizations reported early in the season on the basis of rapid tests were not included as cases until adequate virologic evidence of circulating influenza virus was demonstrated by testing at the state laboratory. Hospitals were requested to submit repeat specimens that tested positive by rapid diagnostic tests to the state laboratory for confirmatory testing by PCR (viral cultures were additionally performed in 2004–05). After approximately half of specimens referred in a given week were confirmed by RT-PCR, hospitals were informed that they no longer needed to refer specimens to the state laboratory; reported hospitalized cases based on positive rapid tests were included. The dates for including reported hospitalized cases based on rapid test results occurred during weeks 51, 50, 6, and 48, respectively, for the 2004–05 through 2007–08 seasons, respectively.

Outpatient surveillance for ILI has been a longstanding component of influenza surveillance at the state and national levels. CDC has suggested that states recruit a minimum of 1 healthcare provider per 250,000 population to report weekly the total number of patients seen and the number of those patients with ILI. During the influenza seasons included in this analysis, Colorado’s volunteer provider–to-population ratio ranged from ≈1 provider per 165,000 persons (2004–05) to 1 provider per 244,000 persons (2007–08 season). The time series from Colorado ILI data (*5*) was qualitatively (timing and relative magnitude of peak) compared with that from reported hospitalized influenza cases to provide a measure of validity.

Characteristics of reported influenza hospitalizations for each season were summarized by percentages and number of reported influenza hospitalizations. Population estimates for 2004–2007 obtained from the Colorado Department of Local Affairs were used to calculate seasonal age-specific rates of influenza-associated hospitalization. Population estimates for the first calendar year of each season (e.g., 2004 for the 2004–05 influenza season) were used to calculate each season’s rates. Data analysis was conducted using SAS version 9.1 (SAS Institute Inc., Cary, NC, USA).

## Results

Influenza hospitalizations were reported from a mean of 44 (range 38–47, 75%) acute care hospitals (licensed for >25 beds during the 4 influenza seasons. The 2006–07 season was notable for having the lowest number of reporting hospitals; however, many of these reported substantially fewer cases compared with the 3 other seasons. Overall, 90% (range 86%–92%) of influenza hospitalizations were reported within 7 days and 68% (range 64%–71%) were reported within 3 days of diagnosis.

The total number of reported influenza hospitalizations varied somewhat across the 4 seasons as did the distribution of selected characteristics of the cases (Table 1). Similar numbers of influenza cases were reported during the 2004–05 (n = 978) and 2007–08 (n = 1,004) seasons; slightly lower numbers were reported during the 2005–06 (n = 848) season. In contrast, only 367 influenza hospitalizations were reported during the 2006–07 influenza season. Similarly, moderate proportions of influenza B hospitalizations were reported during the 2004–05 and 2005–06 seasons (13% for each); a low proportion (3.3%) of influenza B was reported during 2006–07. An unusually high proportion of influenza B (34.2%) was reported among influenza hospitalizations during 2007–08.

**Table 1:** Characteristics of patients hospitalized with influenza, Colorado, USA, 2004–08 influenza seasons*

Characteristics	Influenza season (October 1–May 31), no. (%) patients			
	2004–05	2005–06	2006–07	2007–08
Total recorded cases	978	848	367	1,004
Influenza type				
A	777 (79.45)	699 (82.43)	345 (94.01)	629 (62.65)
B	127 (12.99)	110 (12.97)	12 (3.27)	343 (34.16)
Unknown	74 (7.57)	39 (4.60)	10 (2.72)	32 (3.19)
Age				
<6 mo	64 (6.54)	81 (9.55)	39 (10.63)	79 (7.87)
6–23 mo	72 (7.36)	103 (12.15)	46 (12.53)	78 (7.77)
2–4 y	56 (5.73)	59 (6.96)	27 (7.36)	65 (6.47)
5–17 y	56 (5.73)	72 (8.49)	29 (7.90)	74 (7.37)
18–49 y	140 (14.31)	86 (10.14)	78 (21.25)	180 (17.93)
50–64 y	149 (15.24)	103 (12.15)	39 (10.63)	142 (14.14)
65–79 y	201 (20.55)	169 (19.93)	56 (15.26)	179 (17.83)
>80 y	240 (24.54)	175 (20.64)	53 (14.44)	207 (20.62)
Gender				
M	488 (49.90)	405 (47.76)	186 (50.68)	461 (45.92)
F	485 (49.59)	443 (52.24)	180 (49.05)	517 (51.49)
Unknown	5 (0.51)	0 (0.00)	1 (0.27)	26 (2.59)
Region*				
Western Slope	57 (5.83)	100 (11.79)	31 (8.45)	94 (9.36)
Northern Front Range	122 (12.47)	108 (12.74)	48 (13.08)	121 (12.05)
Denver Metro	550 (56.24)	383 (45.17)	210 (57.22)	520 (51.79)
South Central	36 (3.68)	32 (3.77)	8 (2.18)	14 (1.39)
San Luis Valley	7 (0.72)	8 (0.94)	12 (3.27)	15 (1.49)
Southern Front Range	174 (17.79)	177 (20.87)	48 (13.08)	205 (20.42)
Eastern Plains	32 (3.27)	40 (4.72)	10 (2.72)	35 (3.49)

*Colorado regions can be further divided into counties: Western Slope: Archuleta, Delta, Dolores, Eagle, Garfield, Grand, Gunnison, Hinsdale, Jackson, La Plata, Mesa, Moffat, Montezuma, Montrose, Ouray, Pitkin, Rio Blanco, Routt, San Juan, San Miguel, Summit; Northern Front Range: Larimer, Weld; Denver Metro: Adams, Arapahoe, Boulder, Broomfield, Denver, Douglas, Jefferson; South Central: Chaffee, Clear Creek, Custer, Fremont, Gilpin, Huerfano, Lake, Las Animas, Park, Teller; San Luis Valley: Alamosa, Conejos, Costilla, Mineral, Rio Grande, Saguache; Southern Front Range: El Paso, Pueblo, and Eastern Plains: Baca, Bent, Cheyenne, Crowley, Elbert, Kiowa, Kit Carson, Lincoln, Logan, Morgan, Otero, Phillips, Prowers, Sedgwick, Washington, Yuma.

For 3 of the 4 seasons, the greatest numbers and percentages of influenza hospitalizations were among persons >80 years of age (Table 1). In contrast, the 2006–07 season was noteworthy for lower proportions of cases in the 50–64 y, 65–79 y, and >80 y age groups, and a higher proportion in the 18–49 y age group. By region, overall distributions of cases were fairly similar across seasons.

Distribution of test types was similar across the 4 seasons. Rapid diagnostic tests were the most frequently reported test type (mean 87.3%, range 85.1%– 88.3%) followed by viral culture (mean 5.75%, range 4.8%–7.4%) and DFA (mean 5.7%, range 4.3%–6.9%). PCR was the reported test type for <1.5% of cases in any given season.

**Figure 1:**
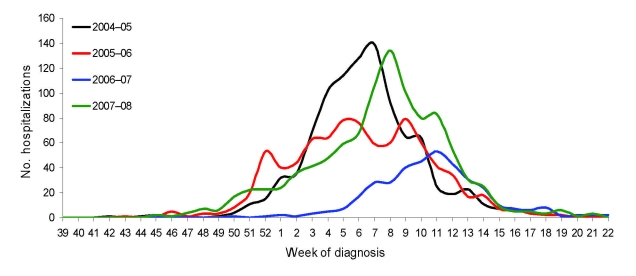
Hospitalized influenza patients in Colorado, USA, by week of diagnosis and influenza season.

The time series of reported influenza hospitalizations for each of the 4 seasons (Figure 1) showed that the 2004–05 and the 2007–08 seasons peaked at almost the same time (weeks 7 and 8, respectively) and with similar magnitude. However, influenza hospitalizations began to increase several weeks earlier during the 2007–08 season with a less steep upslope. In contrast, the 2005–06 season appeared to have 2 peaks of similar magnitude during weeks 5 and 9, and the 2006–07 season exhibited the latest onset (weeks 3–4) and peak (week 11) and the lowest magnitude. By week 15, the time series for all 4 seasons converged at low levels of reported hospitalizations.

Compared with the time series from surveillance for ILI, the timing of influenza hospitalization peaks was quite similar (Table 2). ILI and influenza hospitalizations peaked the same week during the 2004–05 and 2007–08 seasons. The 2005–06 season showed 3 peaks for ILI and influenza hospitalizations (the first was a minor peak; Figure 1); the corresponding time differences were 0, 1, and 2 weeks. During the 2006–07 season, ILI peaked 2 weeks earlier than influenza hospitalizations. The relative magnitudes of peak ILI also corresponded to the relative magnitudes of reported influenza hospitalizations; the lowest magnitude for each occurred during the 2006–07 season.

**Table 2:** Timing of peak activity for influenza hospitalizations and influenza-like illness, Colorado, USA, 2004–08

Category	Influenza season (October 1–May 31)			
	2004–05	2005–06	2006–07	2007–08
Hospitalizations, wk	7	5, 9*	11	8
Influenza-like illness, wk	7	52, 4, 11	9	8

*Smaller initial peak during wk 52.

**Figure 2:**
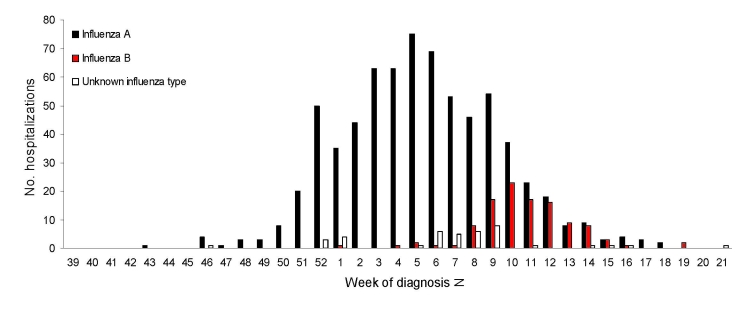
Hospitalized influenza patients in Colorado, USA, by week of diagnosis and influenza type, 2005–06 season.

The time series of hospitalizations stratified by influenza type showed distinctly different patterns among seasons. During the 2004–05 season, influenza A and B peaked at week 7, although the influenza B proportion was relatively small. The 2005–06 season was notable for distinctly separate time courses for influenza A and B (i.e., 2 waves of activity) with influenza A hospitalizations peaking 5 weeks before that for influenza B (Figure 2). The 2006–07 season was mild with minimal influenza B activity. The 2007–08 season was notable for a high proportion of influenza B activity, and the time courses for influenza A and B hospitalizations were superimposed with both peaking at week 8.

**Figure 3:**
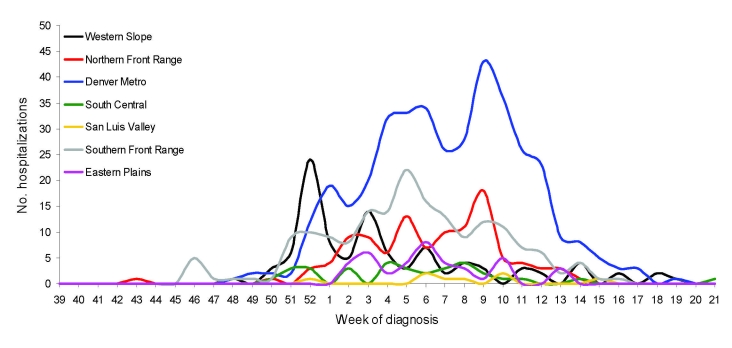
Hospitalized influenza patients in Colorado, USA, by week of diagnosis and region, 2005–06 season.

When stratified by geographic region, the time series of influenza hospitalizations showed peaks that clustered within 2 to 3 weeks for 3 of the 4 seasons. During 2005–06, however, the Western Slope geographic region (west of the Continental Divide) showed a distinct early peak 10 weeks before the Denver metropolitan area; other regions peaked at varying weeks between peak in Western Slope and peak for Denver (Figure 3).

Age group–specific rates of influenza hospitalizations for 3 of the 4 influenza seasons showed characteristic U-shaped distributions, with rates highest for infants <6 months of age and adults >80 years of age (Table 3). Because the 2006–07 season was uncharacteristically mild, lower rates were seen for all age groups, especially for persons >65 years of age, resulting in more of a J-shaped distribution. Children 6–23 months of age, for whom influenza vaccination has been recommended since 2004, had the third highest age group–specific rate of hospitalization during 3 of the 4 seasons (second highest rate during 2006–07). There was no apparent declining trend across the 4 seasons in rates of hospitalizations in this age group; in fact, rates were similar during 2004–05 and 2007–08; fluctuation during the 2 intervening seasons was wide.

**Table 3:** Rates of influenza hospitalizations per 100,000 population, by age group, Colorado, USA, 2004–08

Age group	Influenza season (October 1–May 31), no. cases/100,000			
	2004–05	2005–06	2006–07	2007–08
<6 mo	185.6	234.6	111.8	225.4
6–23 mo	104.3	148.9	66.0	110.5
2–4 y	27.9	28.4	12.8	30.3
5–17 y	6.7	8.6	3.4	8.5
18–49 y	6.2	3.8	3.4	7.8
50–64 y	19.4	12.8	4.6	16.1
65–79 y	59.4	49.0	15.9	49.5
>80 y	214.4	153.6	45.7	174.5
Overall rate (all age groups)	21.2	18.1	7.7	20.6

**Figure 4:**
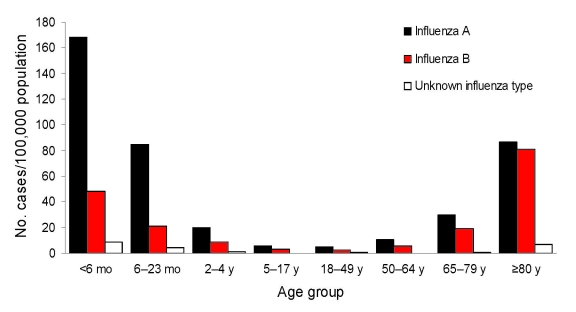
Rates of reported influenza hospitalizations in Colorado, USA, by age group and influenza type, 2007–08 season.

When stratified by influenza type, age group–specific rates for influenza B hospitalizations were greatest for those <6 months and 6–23 months of age during all but the 2007–08 season. In contrast, the 2007–08 season was noteworthy for unusually high rates of influenza B, especially for persons >80 years of age, but also for persons 60–79 years of age. For the >80 years age group, rates of influenza A and B hospitalizations were almost the same, whereas, for infants <6 months of age and children 6–23 months of age, rates of influenza A were approximately 3.5–4× higher than those for influenza B (Figure 4).

## Discussion

This summary of surveillance data from case-based reporting of influenza hospitalizations highlights the similarities and differences among influenza seasons. Each of several characteristics was fairly similar for 3 of the 4 seasons presented (not necessarily the same 3), including the numbers and time course of hospitalizations and age distribution of cases and rates. In contrast, the amount and timing of influenza B activity demonstrated substantial variability. On the basis of the combination of characteristics available from reporting of influenza hospitalizations though, no 2 seasons were entirely the same; 2005–06 had 2 distinct waves of activity (types A and B), 2006–07 was substantially later and milder, and 2007–08 had substantially greater influenza B activity.

Surveillance for influenza hospitalizations during the past 4 seasons in Colorado has substantially added to the state health department’s ability to monitor and describe seasonal influenza. Implementation and maintenance of this surveillance activity has been easily absorbed by the existing influenza surveillance coordinator position and has required no additional resources. Approximately 20% of the surveillance coordinator’s weekly time is devoted to managing and tabulating influenza hospitalization surveillance data. Essential to successful implementation has been acceptability by hospital infection control practitioners statewide, who report almost all of the notifiable diseases diagnosed in hospitals in Colorado.

This surveillance component provides more information about seasonal influenza activity than any other surveillance measure (e.g., ILI) currently in widespread use among states. Influenza hospitalization surveillance provides information regarding the time course (start, peak, end) of seasonal influenza activity, including influenza A and B; the reported case numbers and population-based rates of seasonal influenza by influenza virus type, age group, gender, and geographic region; and a measure of the relative severity of an influenza season compared with previous seasons. Some states conduct surveillance for the numbers of pneumonia and influenza hospital admissions, on a syndromic basis, (*6**,**7*) but this may not necessarily be population based and only provides information on time course and relative severity of influenza activity without the additional information available from laboratory-confirmed, case-based reporting.

The relative rates of influenza A and B, especially at the extremes of age, during 2007–08 were unique among the seasons summarized. Influenza A rates were 3.5–4× higher than influenza B rates for young infants and young children 6 –23 months of age, whereas, for persons >80 years of age, and to a lesser extent persons 65–79 years of age, influenza A and B rates were similar. Since infants <6 months old are not approved to receive influenza vaccine and will not have acquired their own immunity from previous influenza seasons, their rates of hospitalization related to influenza A versus B most closely reflected the epidemiology of circulating influenza viruses, on the basis of prevalence and relative virulence. Low rates of vaccination among infants 6–23 months of age (*8*), for whom influenza vaccination has been recommended since 2004, produce similar relative rates of influenza A and B as for infants <6 months old. In contrast, a high proportion of older adults receive seasonal influenza vaccine (*9*), and influenza type-specific rates of hospitalization in older age groups might reflect protection conferred by vaccination with the current season’s vaccine and possibly cross-protection from previous influenza infection or immunization (*10**,**11*). The 2007–08 influenza vaccine was suboptimally matched to circulating viruses, and estimated vaccine efficacy against the predominant influenza A strain was 58% compared with zero vaccine efficacy against circulating influenza B virus (*12*). Thus, the observed rates of influenza A and B hospitalizations for the older age groups during 2007–08 might reflect partial protection from a moderately effective vaccine against the predominant circulating influenza A virus and no protection from an ineffective vaccine against the predominant circulating influenza B virus.

The validity of influenza hospitalization surveillance as an indicator of seasonal influenza activity was supported by comparison with Colorado ILI surveillance data, which showed good agreement. ILI surveillance can be somewhat challenging to maintain at the state level due to its reliance on providers willing to report data regularly (weekly) for which they are not compensated (hence the range of ratios for participating provider to population during the 4 seasons included). Although ILI surveillance is a longstanding component of seasonal influenza surveillance and has been heavily promoted by CDC, reports of formal evaluation of this surveillance activity are lacking. Colorado hospitalized influenza surveillance data also showed fair agreement with national summary indicators (timing and relative severity) of seasonal influenza for the 4 seasons included. However, this is not the most appropriate comparison because national surveillance data are an aggregate of regional influenza outbreaks that typically vary in time course and intensity.

There are several limitations to these data resulting from surveillance for influenza-related hospitalizations. First, rapid influenza tests, which were the tests used for >85% of the hospitalizations in this report, have suboptimal performance characteristics. The sensitivity of rapid influenza tests is only moderate, more so among adults than children (*13**,**14*). This will result in underestimation of the true rates of influenza hospitalization, to a greater degree for adults than for children, because adults may be admitted for influenza-related complications a number of days after influenza infection when virus is less readily detectible. The positive predictive value of influenza rapid tests is low (and probability of a false-positive test result is high) when the prevalence of circulating influenza virus is low (*15*), which occurs during the early and late parts of the influenza season. Use of influenza rapid tests can result in false identification of the start of seasonal influenza activity based on reported hospitalizations and extend the left tail of the time series curve. To address this issue, the Colorado state health department does not include early season hospitalized influenza cases in official case counts or surveillance data summaries until the prevalence of circulating influenza virus is documented to be adequate based on virologic surveillance by RT-PCR at the state health department laboratory (see Methods).

Second, testing practices can affect ascertainment of hospitalized influenza cases. This is likely more of an issue for adults among whom exacerbation of underlying co-morbidities by influenza might not result in testing for influenza at the time of hospital admission. In 1 study involving retrospective medical records review, a low proportion of adults with a discharge diagnosis of pneumonia had been tested for influenza (*16*).

Third, passive public health surveillance of reportable diseases is known to be incomplete (*17**–**19*). Reporting of influenza hospitalizations as part of passive, case-based notifiable disease reporting is no exception. Some data on completeness of reporting of hospitalized influenza cases from the Denver metropolitan area (approximately half the state’s population) was available from review of statewide hospital discharge data combined with retrospective medical records review as part of a special multisite enhanced influenza surveillance project (*20*). During the 2006–07 season, completeness of reporting of adult hospitalized patients with positive test results in the medical record was estimated to be 65% (*16*) and 70% for pediatric cases (Colorado Department of Public Health, unpub. data). For the 2007–08 season, estimated completeness of reporting was 75% for adult cases and 66% for pediatric cases (Colorado Department of Public Health, unpub. data). Thus, it seems unlikely that variable completeness of reporting between pediatric and adult cases or across seasons is the main factor contributing to variation in the relative age group–specific rates of hospitalized patients with influenza. Multiple other factors that might contribute to the observed epidemiologic pattern include virulence of seasonal circulating viruses, host immunity from previous seasons, and protection afforded by each season’s vaccine.

Despite these limitations that undoubtedly resulted in underascertainment of influenza-related hospitalizations, to a greater extent for adults than children, surveillance for influenza hospitalizations can contribute useful information for public health monitoring of seasonal influenza activity. The numbers of cases and rates derived from passive reporting of hospitalized influenza cases should be viewed as a minimum estimate, especially for adults. Incomplete case ascertainment and reporting should have little effect on monitoring the time course of hospitalizations for patients with influenza. As is true for passive surveillance systems in general, assessing the relative severity of a given influenza season should still be valid as long as surveillance methods and system performance (i.e., completeness of reporting) remain relatively unchanged over time.

In conclusion, case-based surveillance for laboratory-confirmed influenza in hospitalized patients provides multiple useful population-based measures of seasonal influenza activity that focus on more severe illness attributed to influenza. Influenza hospitalization surveillance can also contribute to better characterization of the epidemiology of influenza across seasons. If more state health departments implemented case-based surveillance for influenza hospitalizations, the aggregated data could comprise a useful contribution to national influenza surveillance. Surveillance for influenza hospitalizations might also contribute to state surveillance capacity in preparation for an influenza pandemic as well as help target vaccination programs for seasonal influenza.

## References

[1] Thompson WW, Shay DK, Weintraub E, Brammer L, Cox N, Anderson LJ, Mortality associated with influenza and respiratory syncytial virus in the United States. JAMA. 2003;289:179–86. 10.1001/jama.289.2.179 12517228

[2] Thompson WW, Shay DK, Weintraub E, Brammer L, Bridges CB, Cox NJ, Influenza-associated hospitalizations in the United States. JAMA. 2004;292:1333–40. 10.1001/jama.292.11.1333 15367555

[3] Centers for Disease Control and Prevention. Influenza [cited 2008 26 Jul]. Available from http://www.cdc.gov/flu

[4] Centers for Disease Control and Prevention. Surveillance for laboratory-confirmed, influenza-associated hospitalizations—Colorado, 2004–05 influenza season. MMWR Morb Mortal Wkly Rep. 2005;54:535–7. 15931157

[5] Colorado Department of Public Health and Environment. Summaries of previous influenza seasons [cited 2008 Oct 26]. Available from http://www.cdphe.state.co.us/dc/Influenza/index.html

[6] Louie JK, Schnurr DP, Guevara HF, Honarmand S, Cheung M, Cottam D, Creating a model program for influenza surveillance in California: results from the 2005–2006 influenza season. Am J Prev Med. 2007;33:353–7. 10.1016/j.amepre.2007.05.008 17888862

[7] Hadler JL, Siniscalchi A, Dembek Z. Hospital admissions syndromic surveillance—Connecticut, October 2001–June2004. In: Syndromic surveillance: reports from a national conference, 2004. MMWR Morb Mortal Wkly Rep. 2005;54(Suppl):169–73. 16177710

[8] Centers for Disease Control and Prevention. Influenza vaccination coverage among children aged 6–23 months—United States, 2006–07 influenza season. MMWR Morb Mortal Wkly Rep. 2008;57:1039–43. 18818583

[9] Centers for Disease Control and Prevention. State-specific influenza vaccination coverage among adults—United States, 2006–07 influenza season. MMWR Morb Mortal Wkly Rep. 2008;57:1033–9. 18818582

[10] Nguyen HH, Zemlin M, Ivanov II, Andrasi J, Zemlin C, Vu HL, Heterosubtypic immunity to influenza A virus infection requires a properly diversified antibody repertoire. J Virol. 2007;81:9331–8. 10.1128/JVI.00751-07 17567700 PMC1951409

[11] Quan FS, Compans RW, Nguyen HH, Kang SM. Induction of heterosubtypic immunity to influenza virus by intranasal immunization. J Virol. 2008;82:1350–9. 10.1128/JVI.01615-07 18032492 PMC2224423

[12] Centers for Disease Control and Prevention. Interim within-season estimate of the effectiveness of trivalent inactivated influenza vaccine—Marshfield, Wisconsin, 2007–08 influenza season. MMWR Morb Mortal Wkly Rep. 2008;57:393–8. 18418344

[13] Hurt AC, Alexander R, Hibbert J, Deed N, Barr IG. Performance of six influenza rapid tests in detecting human influenza in clinical specimens. J Clin Virol. 2007;39:132–5. 10.1016/j.jcv.2007.03.002 17452000

[14] Ruest A, Michaud S, Deslandes S, Frost EH. Comparison of the Directigen flu A+ B test, the QuickVue influenza test, and clinical case definition to viral culture and reverse transcription-PCR for rapid diagnosis of influenza virus infection. J Clin Microbiol. 2003;41:3487–93. 10.1128/JCM.41.8.3487-3493.2003 12904343 PMC179849

[15] Grijalva CG, Poehling KA, Edwards KM, Weinberg GA, Staat MA, Iwane MK. Accuracy and interpretation of rapid influenza tests in children. Pediatrics. 2007;119:e6–11. 10.1542/peds.2006-1694 17200259

[16] Sadlowski J, Gershman K, Burnite S, Conroy A, Juhl A, Use of hospital discharge data to assess completeness of reporting of adult influenza-associated hospitalizations, Colorado, 2006–07 [abstract]. Presented at: 2008 International Conference on Emerging Infectious Diseases; March 16–19, 2008; Atlanta, GA, USA [cited 2009 Apr 6]. Available from http://www.cdc.gov/EID/content/14/3/ICEID2008.pdf

[17] Vogt RL, Larue D, Klaucke DN, Jillson DA. Comparison of active and passive surveillance systems of primary care providers for hepatitis, measles, rubella, and salmonellosis in Vermont. Am J Public Health. 1983;73:795–7. 10.2105/AJPH.73.7.795 6859365 PMC1650894

[18] Thacker SB, Redmond S, Rothenberg R, Spitz SB, Choi K, White MC. A controlled trial of disease surveillance strategies. Am J Prev Med. 1986;2:345–50. 3453201

[19] Sacks JJ. Utilization of case definitions and laboratory reporting in the surveillance of notifiable communicable diseases in the United States. Am J Public Health. 1985;75:1420–2. 10.2105/AJPH.75.12.1420 4061715 PMC1646445

[20] Schrag SJ, Shay DK, Gershman K, Thomas A, Craig AS, Schaffner W, Multisate surveillance for laboratory-confirmed, influenza-associated hospitalizations in children 2003–04. Pediatr Infect Dis J. 2006;25:395–400. 10.1097/01.inf.0000214988.81379.71 16645501

